# Juxtaventricular and periventricular white matter hyperintensities (WMH) are associated with cognitive dysfunction in Patients with Alzheimer’s Disease

**DOI:** 10.1192/j.eurpsy.2024.496

**Published:** 2024-08-27

**Authors:** J. H. Park, H. J. Yang, J. M. Song

**Affiliations:** ^1^Psychiatry, Jeju National University School of Medicine, Jeju National University Hospital; ^2^Psychiatry, jeju Medical Center, Jeju, Korea, Republic Of

## Abstract

**Introduction:**

White matter hyperintensities (WMH) is common among the elderly. WMH are associated with accelerated cognitive dysfunction and increased risk for Alzheimer`s disease (AD). Although WMHs play a key role in lowering the threshold for the clinical expression of dementia in AD-related pathology, the clinical significance of their location is not fully understood.

**Objectives:**

The aim of this study was twofold: 1) To investigate the quantitative association between WMH and cognitive function in AD; 2) To investigate whether there is any difference in the association between subclassified WMH and cognitive function in AD.

**Methods:**

A total of 171 patients with AD underwent clinical evaluations including volumetric brain MRI study and neuropsychological tests using the CERAD-K neuropsychological assessment battery. WMH volume was calculated using automated quantification method with SPM and MATLAB image processing software. According to the distance from the lateral ventricular surface, WMH within 3 mm, WMH within 3-13 mm, and WMH over 13 mm were classified as juxtaventricular WMH (JVWMH), periventricular WMH (PVWMH) and deep WMH (DWMH), respectively. WMH volume data was logarithmically transformed because it was right-skewed.

**Results:**

WMH volume in AD was 20.7 ± 18.2 ml. Total WMH volume was associated with poor performance in categorical verbal fluency test (p = 0.008) and word list memory test (p = 0.023). JVWMH volume was associated with poor performances on categorical verbal fluency test (p = 0.013) and forward digit span test (p = 0.037). PVWMH volume was associated with poor performances on categorical verbal fluency test (p = 0.011) and word list memory test (p = 0.021), whereas DWMH volume showed no association with cognitive tests. Total WMH and PVWMH volume were also related to Clinical Dementia Rating scale sum of boxes score (p=0.022).

**Image:**

**Figure:**
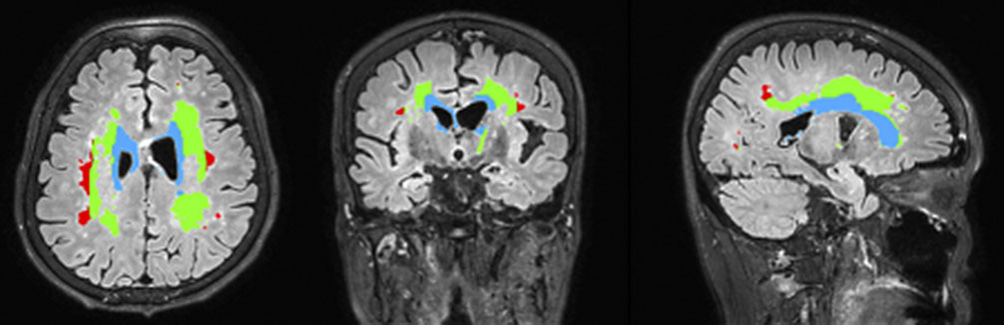

**Conclusions:**

Greater JVWMH and PVWMH are related with concurrent impairments in semantic memory and frontal function independent of the hippocampal volume. However, DWMH volume is not associated with any cognitive function. Only PVWMH among subclassified WMH are related to the severity of AD.

**Disclosure of Interest:**

None Declared

